# Small Molecule Inhibitors of White Spot Syndrome Virus: Promise in Shrimp Seedling Culture

**DOI:** 10.3390/ijms22073450

**Published:** 2021-03-26

**Authors:** Lei Liu, Li-Peng Shan, Yan Zhou, Jiong Chen

**Affiliations:** 1 State Key Laboratory for Managing Biotic and Chemical Threats to the Quality and Safety of Agro-products, Ningbo University, Ningbo 315211, China; liulei2@nbu.edu.cn (L.L.); shanlipeng326@126.com (L.-P.S.); a834069482@126.com (Y.Z.); 2 Laboratory of Biochemistry and Molecular Biology, School of Marine Sciences, Meishan Campus, Ningbo University, Ningbo 315832, China; 3 Key Laboratory of Applied Marine Biotechnology of Ministry of Education, Meishan Campus, Ningbo University, Ningbo 315832, China

**Keywords:** *Litopenaeus vannamei*, white spot syndrome virus, continuous immersion, coumarin, horizontal transmission

## Abstract

Rapid production of prawn (*Litopenaeus vannamei*) under artificial pressure can result in a series of obvious challenges and is vulnerable to serious losses related to aquatic environmental issues and the unrestrained outbreak of white spot syndrome (WSS). However, to date, there are no therapeutic strategies to contain the spread of the virus. Here, we synthesized 27 coumarin derivatives and evaluated their anti-white spot syndrome virus (WSSV) activity in *L. vannamei* larvae. We demonstrated that electron-withdrawing and electron-giving substituent groups play an important role in reducing toxicity and WSSV replication, respectively. Two coumarin **C2** (2-amino-5-oxo-4-(p-tolyl)-4H,5H-pyrano[3,2-c]chromene-3-carbonitrile) and **C7** (2-amino-4-(4-chlorophenyl)-5-oxo-4H,5H-pyrano[3,2-c]chromene-3-carbonitrile) were regarded as the most promising anti-WSSV compounds with maximum antiviral response <5% and median effective concentration <10 mg/L. The mortality of WSSV-infected larvae decreased by more than 60% after exposure to **C2** and **C7**. With continuous immersion of **C2** and **C7** exchange, the mortality further decreased to 40% at 120 h. Additionally, **C2** and **C7** are the relatively stable in aquacultural water, making these agents suitable for use in inhibiting WSSV horizontal transmission in static aquaculture systems. These results showed the marked advantages of using **C2** and **C7** in the shrimp industry, and suggest that they hold potential for the treatment and prevention of WSSV infection in shrimp seedling culture.

## 1. Introduction

Aquacultural food production contributes to employment, a diversity of income strategies, and human nutrition and health. The long-term production and supply of both wild-capture fisheries and aquaculture are projected to be enhanced in the next few decades to meet global demands for aquatic food. Among the main species of aquaculture, seawater and low-salinity brackish-water shrimp culture farming has become a global industry as one of the most economically productive activities, given that the total global production of shrimp is more than 4.7 million tons in 2019, based on the report of Global Aquaculture Alliance (https://www.aquaculturealliance.org/, accessed on 25 March 2021).

Nevertheless, rapid shrimp production under artificial pressure can be prone to aquatic ecosystem challenges. In particular, the shrimp industry is vulnerable to serious losses associated with the emergence and outbreak of viral diseases because of the lack of efficacious treatments or preventative measures [1]. White spot syndrome (WSS) is the most serious viral disease in the shrimp-cultivation industry and is caused by WSS virus (WSSV) [2,3,4]. Since it was first reported in 1992 in the south of China [5], WSS in virtue of global trade transporting large quantities has rapidly spread to all shrimp-farming regions, including Asia [6], America [3], Europe [7], Africa [8], the Middle East [8], and Australia [9]. WSSV is an enveloped, double-stranded DNA virus classified as the only member of the genus *Whispovirus*, family *Nimaviridae*, and it infects various crustaceans [10]. It is particularly lethal to penaeid shrimps, causing up to 90‒100% mortality within 3~10 days [11]. Consequently, WSSV is a notifiable pathogen on the list of the World Organization for Animal Health [4]. In addition to the lack of efficacious treatments or preventative measures, complex routes of infection transmission and its broad host range also make its eradication from affected ponds difficult [12], which further intensifies the potential damage. Therefore, prophylactic or therapeutic agents are necessary for development and application against WSSV infection in the shrimp industry.

Various natural compounds from herbal products or plant extracts have broad pharmacological application prospects due to their antiviral activities [13,14,15]. One class of natural compounds that has been extensively studied is coumarins, which are phytochemicals occurring naturally in plants, bacteria, and fungi. Coumarins target various cellular pathways, inhibiting the growth and replication of viruses [16,17,18,19,20]. In addition, a coumarin (benzo-α-pyrone) with an oxygen heterocycle and the parent chemical structure is considered to be the leading compound for further chemical synthesis or structural modifications [16]. Earlier synthetic coumarins with a variety of pharmacophoric groups at the C-3, C-4, and C-7 positions have been screened intensively for antiviral effects, based on the correlation between structural characteristics and biological activities [21]. In addition to its application in human viral diseases, it has the advantages of being stable, soluble, and of low molecular weight, without any adverse side effects and toxicity, making coumarin derivatives candidate drugs for use against aquatic viruses [18,19,22,23,24], including WSSV.

In an attempt to address the urgent need for therapeutics for prophylaxis and treatment of WSS, in this study we designed and synthesized a series of novel 7-hydroxycoumarin derivatives, which have nitrogen-containing heterocyclic structures with different electron-withdrawing and electron-donating groups. We identified their anti-WSSV effects in shrimp larvae by determining the medium lethal concentration (LC_50_; mg/L), median effective concentration (EC_50_; mg/L), survival rate, and continuous immersion and horizontal transmission assays. A further preliminarily study with preincubated WSSV suggested that the active coumarins exerted additional damaging effects on the virus, contributing to induction of an innate immune response in shrimp. The present study thus suggests that the anti-WSSV coumarins have a significant protective effect on shrimp larvae and can be advantageous to the shrimp breeding industry.

## 2. Results and Discussion

The production of seawater and low-salinity brackish-water shrimp has grown from 1325 to 4,875,793 metric tons over the past approximately 70 years [1]. As the main species involved in the commercial shrimp sector, *Litopenaeus vannamei* accounted for 80% of global production in 2015. WSSV caused a significant loss in the shrimp sector, particularly in the culture of shrimp seedlings [25]. Traditional measures to control WSSV, including environmental management, specially formulated diets, and vaccines, are costly and practically infeasible [26]. Consequently, prophylactic or therapeutic agents need to be developed for application against WSSV infection in the shrimp industry.

### 2.1. Acute Toxicity and Anti-WSSV Activity of a Total of the 27 Coumarin Derivatives

Prior to evaluation of the anti-WSSV activity of coumarins, the safety doses for larvae needed to be determined. The solubility of coumarin C was lower than that of the other two groups (coumarins **B1**–**9** and **C1**–**9**), indicating that coumarin derivatives **C2**, **C5**, **C7**, and **C8**, at the tested concentration of 16 mg/L, were insoluble in aquacultural water at 28 °C;this may be related to the low solubility of malononitrile. No signs of toxicity were observed at the saturation concentration after 72 h of exposure. Under this premise, the data indicated that the overall toxicity of coumarin **C** was also lower than that of coumarin **A** and **B**, which was particularly significant in terms of the four electron-withdrawing halogen and -NO_2_ groups. As shown in Table 1, the **R^1^** substituent group markedly increased the toxicity of 4-phenyl-3,4-dihydro-2H-chromeno [4,3-d] pyrimidine-2,5(1H)-dione (**A1**). In contrast, the electron-withdrawing group was more toxic than the electron-donating group, with the LC_50_ of **A5**‒**A8** being <5 mg/L at 72 h. Furthermore, **A3** had the strongest electron-donating group (-OCH_3_; LC_50_ = 21.113 mg/L), while **A8** had the strongest electron-withdrawing group (-NO_2_; LC_50_ = 1.614 mg/L) and constituted the extremes of the toxic range. Although the **R^1^** substituent group markedly reduced the toxicity of the index compound from coumarin **A**, a similar order of electron donating ability occurred in coumarin **B**, suggesting that the order of toxicity was **B3** (LC_50_ = 41.706 mg/L) < **B2** < **B9** < **B5** < **B6** < **B7** < **B8** (LC_50_ = 3.057 mg/L). Based on preliminary analysis of the structure-activity relationship (SAR) of similar spatial structures with different substituents of electron-donating and electron-withdrawing groups to coumarins **A1**, **B1**, and **C1**, we considered that electron-withdrawing groups could reduce the toxicity of compounds; this provides a basis for further research.

**Figure 1:**
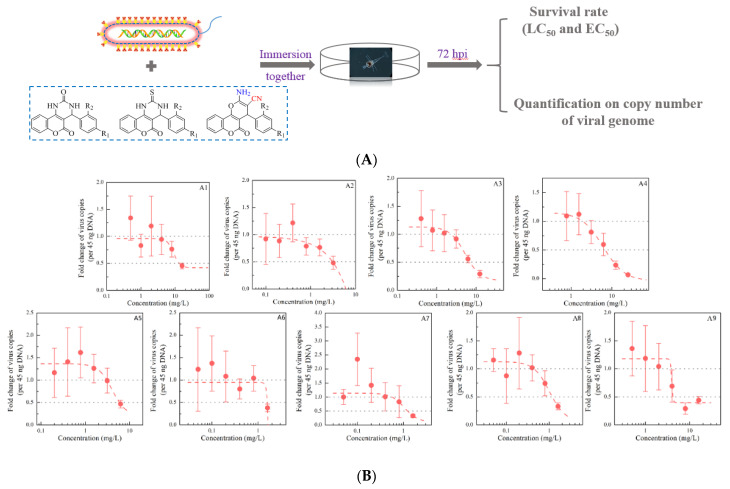

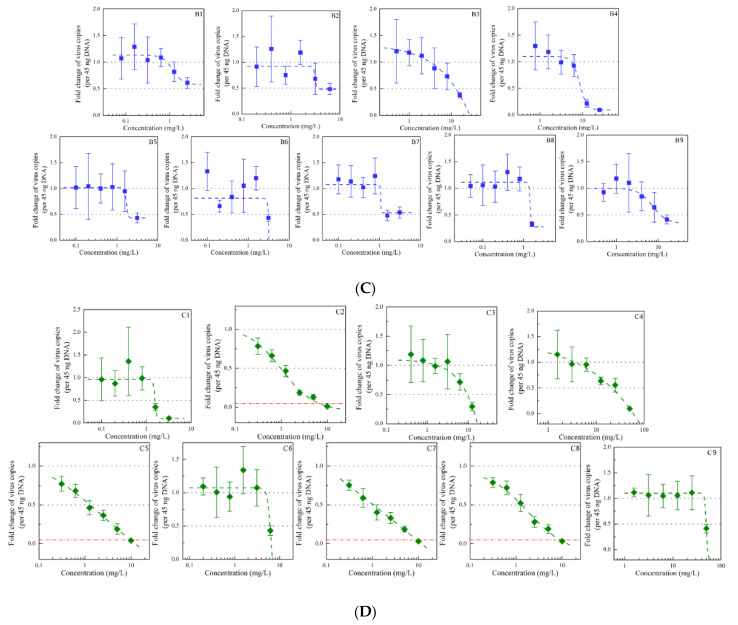
The percent inhibition of the 27 coumarin derivatives on white spot syndrome virus (WSSV) replication in shrimp larvae is shown. (**A**) Schematic diagram describing the workflow. (**B**–**D**) Six-point dose-response curves for antiviral activity of coumarins are examined. The percent inhibition on quantification on copy number of viral genome is analyzed by RT-qPCR. Each value is represented as the mean ± SD normalized to values for no treatment.

For the 27 coumarins, we evaluated the antiviral effects primarily by establishing the maximum antiviral response (MAR) and EC_50_ value. After a preliminary screening, a six-point dose-response S-shaped curve was plotted for the anti-WSSV effect of each compound versus the copy number of the viral genome in virus-infected larvae (Figure 1). The inhibitory efficiency of representative active coumarins **A4**, **B4**, **C2**, **C4**, **C5**, **C6**, **C7**, and **C8** exceeded 90%. The order of protection, based on the EC_50_ value, showed a potency of **C7** < **C2** < **C8** < **C6** < **C5** < **A4** < **B4** < **C4**, suggesting that the activity of coumarins with electron-withdrawing substituents was superior to that of coumarins with electron-donating substituents, except for **C2**. Among three initial compounds (**A1**, **B1,** and **C1**), **C1** showed the most potent antiviral activity against WSSV infection with EC_50_ of 2.903 (2.635~3.251) mg/L and MAR of 10.170%. Indeed, the antiviral activity of coumarin **C** was significantly improved by group modification at the same site. For coumarins **A** and **B**, there were six compounds exhibiting low EC_50_ values (<10 mg/L), including **A2**, **A5**, **A6**, **A8**, **B5**, and **B8**. However, their poor MAR values implied that they did not inhibit WSSV replication in shrimp larvae and, thus, could not provide effective protection, which was largely dependent on low biosafety concentrations. The above results illustrated that electron-withdrawing groups at the **R^1^** substituent improved the biological activity of coumarins. Based on a comprehensive comparison between MAR and EC_50_ value, coumarin **C2** and **C7** were chosen for the further study. Remarkably, the present results complement our earlier findings that coumarins within six and eight carbon atoms length of linker increase toxicity on aquatic cells, and methylimidazole or benzimidazole plays a key role in the antiviral activity in the same length of the linker group [21].

### 2.2. Anti-WSSV Activity of C2 and C7 in Shrimp Larvae

**Figure 2:**
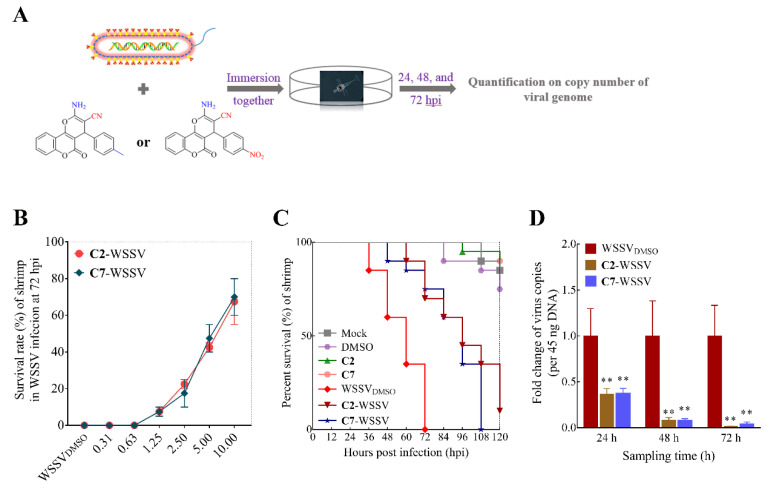
**C2** and **C7** inhibited WSSV infection in shrimp larvae. (**A**) Schematic diagram describing the workflow. (**B**) The survival rate of WSSV-infected shrimp was analyzed in 0.31 to 10 mg/L treatments at 72 hpi. (**C**) Survivorship curve of WSSV-infected shrimp was analyzed in **C2** and **C7** up to 10 mg/L treatment. (**D**) Quantification on copy number of viral genome was tested by exposure to **C2** and **C7** up to 10 mg/L treatment at 24, 48, and 72 hpi, respectively. The virus-infected shrimps were immersed with the compounds and DMSO at the dose 0.2‰ (*v/v*). Each value is represented as the mean ± SD normalized to values for no treatment. The *p* value for each study was determined by Student’s *t* tests. ** *p* < 0.01.

For in vivo study of co-incubation, **C2** and **C7** was separately incubated with WSSV and shrimp larvae. The results showed that the mortality rate of the virus-infected shrimp gradually decreased with the increase in compound concentrations (ranging from 0.31 to 10 mg/L), and the antiviral trend between **C2** and **C7** was consistent (Figure 2B). Specifically, the percentage of survival rate in WSSV-infected larvae at 72 h post-infection (hpi) was significantly enhanced, by more than 65%, after exposure to both **C2** and **C7** at a concentration of 10 mg/L (Figure 2C). Correspondingly, the copy number of the viral genome in sick or dead shrimp homogenates was down-regulated by **C2** and **C7** treatments at 24, 48, and 72 hpi, with WSSV loads sometimes being actually lower in surviving shrimp (Figure 2D).

Invertebrates mainly rely on the innate immune response to resist the invasion of bacteria and viruses due to a lack of adaptive immunity [27]. With a view to achieving protection or therapy to control WSSV infection, there has been a growing interest in natural products because of their ability to stimulate the immune system [28]. For example, five methanol extracts of Indian plants were mixed into shrimp feed to increase the survival rate of WSSV-infected black tiger shrimp [29]. Three chromatographic fractions from the ethanolic extract of *Pongamia pinnata* leaves incorporated into a completely formulated feed also improved the anti-WSSV capability of shrimp [30]. However, it is not practical for the culture of shrimp seedlings, as larvae are more vulnerable to WSSV infection because of an incomplete immune system. Moreover, the component complexity and cost-effectivity of these strategies remain limitations of these approaches [28]. Thus, naturally occurring biological compounds have been isolated and purified from plants or synthesized as potential antiviral agents [19,21,24,31,32,33]. For example, epigallocatechin gallate is a promising therapeutic agent against grass carp reovirus (GCRV) infection by blocking its binding to laminin receptors [34]; saikosaponin D efficiently suppressed spring viremia of carp virus (SVCV) replication in vivo, prolonging the survival of zebrafish and common carp [35]. Compared to the anti-WSSV plant extracts described above, immersion in **C2** and **C7** improved the survival rate of WSSV-infected *L. vannamei* larvae by blocking WSSV replication, indicating their potential application in the culture of shrimp seedlings. The previous study showed that geniposidic acid (GPA) from the ethanol extracts of *Eucommia ulmoides* Oliver suppress WSSV infection in vitro and in vivo [32]. By contrast, application amount of **C2** and **C7** was lower than GPA, and more convenient by immersion. Additionally, numerous coumarins have been evaluated for inhibition against human immunodeficiency virus (HIV) replication, such as 4-hydroxycoumarin analogue warfarin and furanocoumarin imperatorin [36,37,38]. Several promising coumarins, including imidazopyridine-coumarin, purine-coumarin, and benzoxazole-coumarin, have shown substantial anti-hepatitis virus activity [39]. Therefore, coumarin is also regarded as an advantageous structure for the design of novel antiviral agents with high affinity and specificity to various molecular targets.

### 2.3. Pre-Exposure of Antiviral Compounds and Preincubation of WSSV Infection

**Figure 3:**
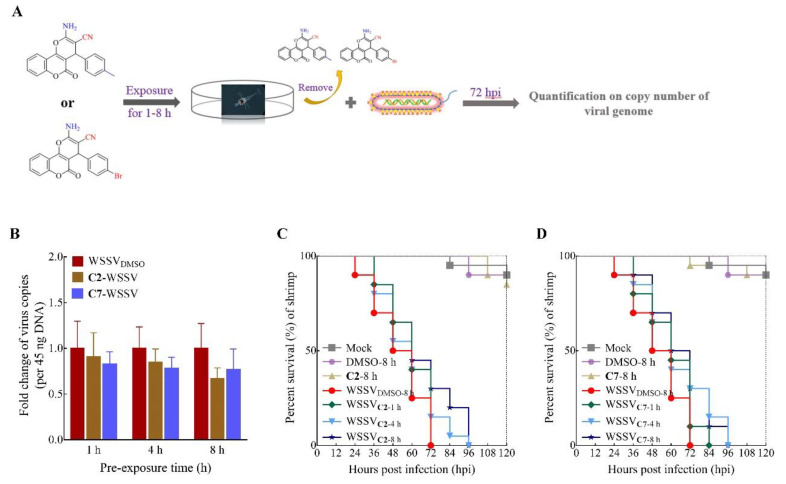
Effect of **C2** and **C7** pre-exposure was evaluated on WSSV infection. (**A**) Schematic diagram describing the workflow. (**B**) Quantification on copy number of viral genome in shrimp larvae was tested in **C2** and **C7** pre-exposure to larvae for 1, 4, and 8 h, followed by WSSV infection. (**C**,**D**) Survivorship curve of WSSV-infected shrimp was analyzed in **C2** and **C7** up to 10 mg/L pre-exposure treatment. Each value is represented as the mean ± SD normalized to values for no treatment. The *p* value for each study was determined by Student’s *t* tests.

In addition to co-incubation of WSSV and compounds, we explored the preventive effects of **C2** and **C7** against virus infection. Unexpectedly, slight changes in the copy number of the viral genome between treatments with the virus alone and compound treatments were observed when larvae were exposed to **C2** and **C7** directly for up to 8 h prior to WSSV infection (Figure 3B). Nevertheless, **C2** or **C7** still delayed shrimp death to 96 hpi, while there was a 100% mortality rate by 72 h after WSSV infection in the absence of treatment (Figure 3C,D), which suggested that unmetabolized and low doses of the compounds in larvae could also inhibit virus replication to some extent.

**Figure 4:**
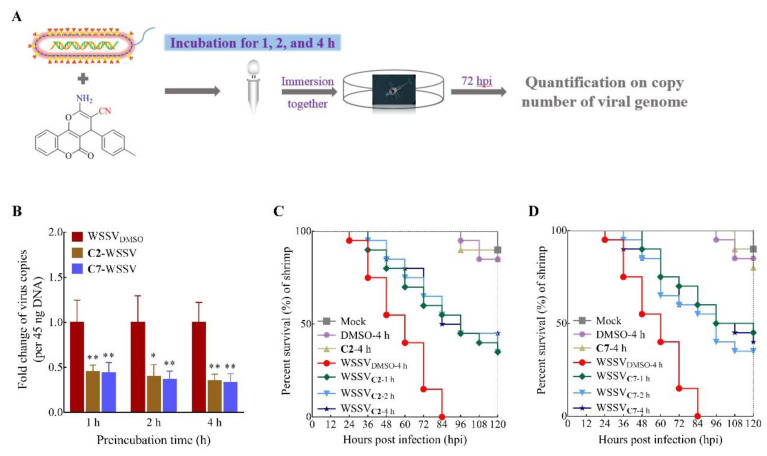
Antiviral activity of **C2** and **C7** was evaluated on preincubation with WSSV. (**A**) Schematic diagram describing the workflow. (**B**) Quantification on copy number of viral genome in shrimp larvae was tested in **C2** and **C7** preincubation with WSSV for 1, 2, and 4 h, followed by infection to larvae. (**C**,**D**) Survivorship curve of WSSV-infected shrimp was analyzed in **C2** and **C7** at the concentration of 10 mg/L in 1, 2, and 4 h preincubation. Each value is represented as the mean ± SD normalized to values for no treatment. The *p* value for each study was determined by Student’s *t* tests. ** *p* < 0.01; * *p* < 0.05.

To address the negative effects of **C2** and **C7** during the WSSV infection cycle, the virus was pre-incubated with the compounds for 1–4 h prior to infection of larvae (Figure 4).The results showed a suppressed WSSV infection (Figure 4B) and increased the survival rate by up to 40% (Figure 4C,D). Based on the findings of previous studies [17,33,40], we consider that **C2** and **C7** may have a direct influence on the infectivity of WSSV virion particles, rather than on the host’s receptor protein, to block virus infection in the early stage. This therefore provides insight into the properties of coumarin products required for anti-WSSV therapeutic inhibitors and for vaccine development.

### 2.4. The Relative Stability of C2 and C7 in Aquacultural Water

**Figure 5:**
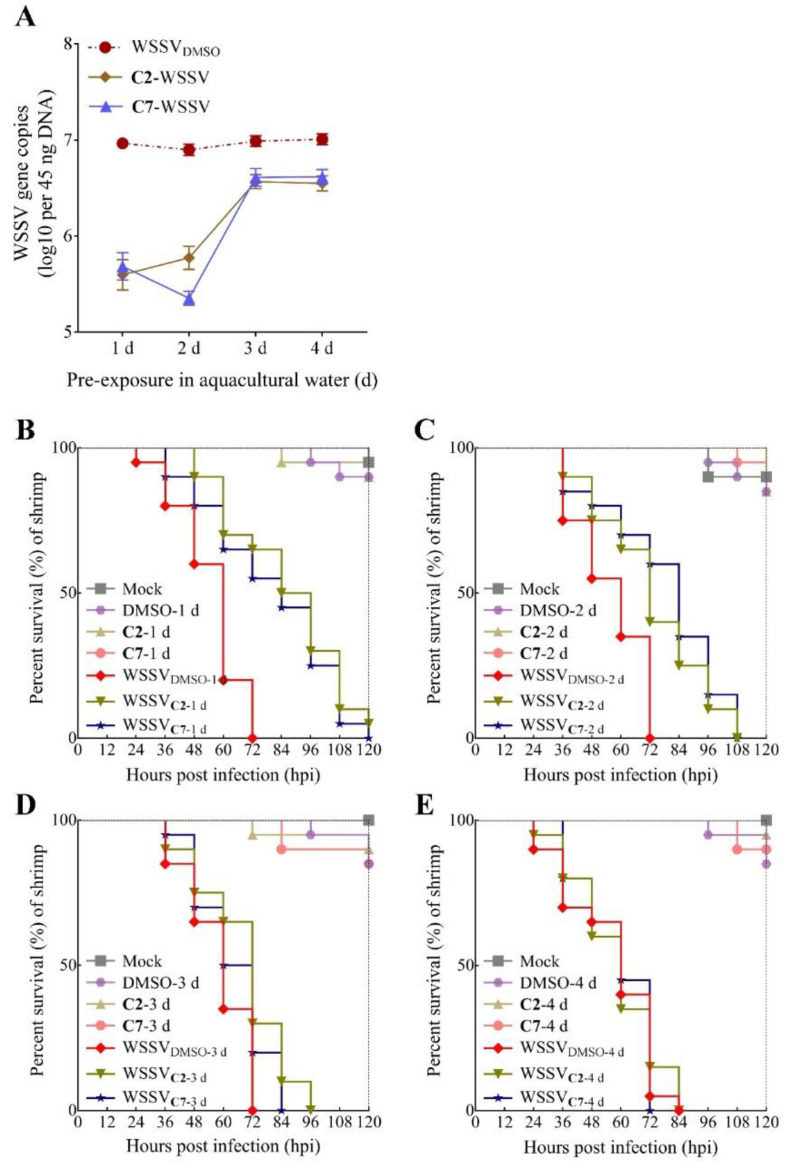
Analysis on the related stability of **C2** and **C7** in aquacultural water at 28 °C. (**A**) Quantification on copy number of viral genome in shrimp larvae was tested in **C2** and **C7** for different water samples by RT-qPCR. Each value is represented as the mean ± SD normalized to values for no treatment. (**B**–**E**) Survivorship curve of WSSV-infected shrimp was analyzed in **C2** and **C7** at the concentration of 10 mg/L in 1, 2, 3, and 4 d water samples.

**C2** and **C7** were added to aquacultural water, at 28 °C, containing a certain amount of organic material. The half-life of **C8** has previously been found to be between 1 and 2 days at 15 °C in aquacultural and sterilized deionized water [33]. In the present study, **C2** and **C7** showed a similar stability in a shrimp aquacultural environment at 28 °C, where significant degradation occurred between 2 and 3 days, because there was significantly enhanced WSSV replication in vivo at 72 hpi (Figure 5A). Additionally, **C2** and **C7** added to water samples had a calculated inhibitory half-life of 2.3 (*R*^2^ = 0.88) and 2.4 (*R*^2^ = 0.83) days, respectively, when a nonlinear regression, best-fit line was applied. More evidence of water stability was reflected in the finding that 100% mortality in compound-treated water samples occurred at the earlier time points: 120 hpi for 1 day (Figure 5B), 108 hpi for 2 days (Figure 5C), 96 hpi for 3 days (Figure 5D), and 84 hpi for 4 days (Figure 5E). Compared to other antiviral compounds that have been reported in aquaculture, such as LJ001 and LJ025 (less than 2 days) [40], half-life of coumarins appear to be more stable in aquacultural water. These results demonstrate that **C2** and **C7** are relatively stable compounds under the complex shrimp aquacultural conditions.

### 2.5. The Inhibition of Continuous Immersion of Compound Exchanges on WSSV Replication in Larvae

**Figure 6:**
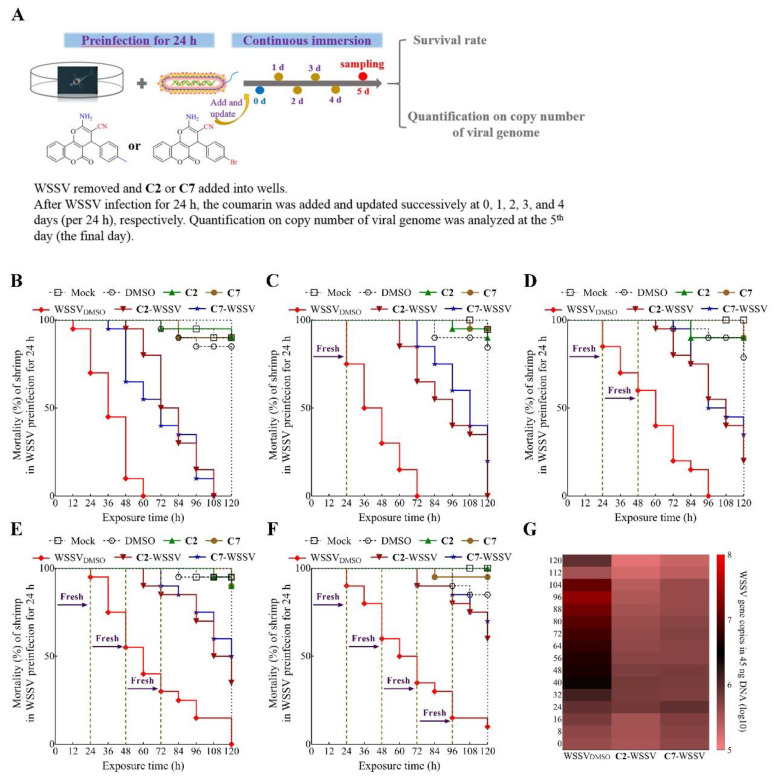
Continuous immersion of **C2** and **C7** exchange provides a strong protection on shrimp larvae against WSSV infection. (**A**) Schematic diagram describing the workflow. (**B**–**F**) Aquacultural water or fresh **C2** and **C7** medium were exchanged at 0, 1, 2, 3, and 4 days, respectively. Survivorship curves of WSSV-infected shrimp was analyzed. (**G**) The heat map indicated that quantification on copy number of viral genome was tested in every 8 h. Each value is represented as the mean ± SD normalized to values for no treatment.

The short half-life of antiviral compounds in water poses their main limitation for use in aquaculture, as it necessitates periodic exchange of the treated water to eliminate the virus threat. Based on the stability of **C2** and **C7** described above, we hypothesized that continuous immersion in coumarin with water exchange could better eliminate the virus in the shrimp and could provide stronger protection for shrimp larvae. To simulate the real infection environment, the larvae were pre-infected with WSSV for 24 h prior to exposure to **C2** and **C7** at the concentration of 10 mg/L. As shown in Figure 6B, the survival rate increased with coumarin treatment to 50% versus 0% in untreated but WSSV-infected larvae at 60 h after exposure. Under the same infection conditions, the survival rate gradually increased from 0% to more than 60% at 120 h when **C2** and **C7** were exchanged one to four times within a 24-h period (Figure 6C–F). It is worth noting that continuous water exchange also helps to improve the survival rate, and this procedure is necessary for shrimp larvae to eliminate excess organic pollutants [41]. Therefore, effective high-frequency water exchange can assist farmers in reducing the risks involved in shrimp aquaculture. Other data, based on monitoring every 8 h, showed that WSSV proliferation in live shrimp was gradually enhanced in the absence of exposure to **C2** and **C7** (Figure 5G). As shrimp at the seedling stage are often exposed to a variety of pathogens [42], including WSSV, during water exchanges, we consider that continuous immersion of **C2** and **C7** can provide vital protection to larvae.

### 2.6. The Effect of C2 and C7 on the Horizontal Transmission of WSSV

**Figure 7:**
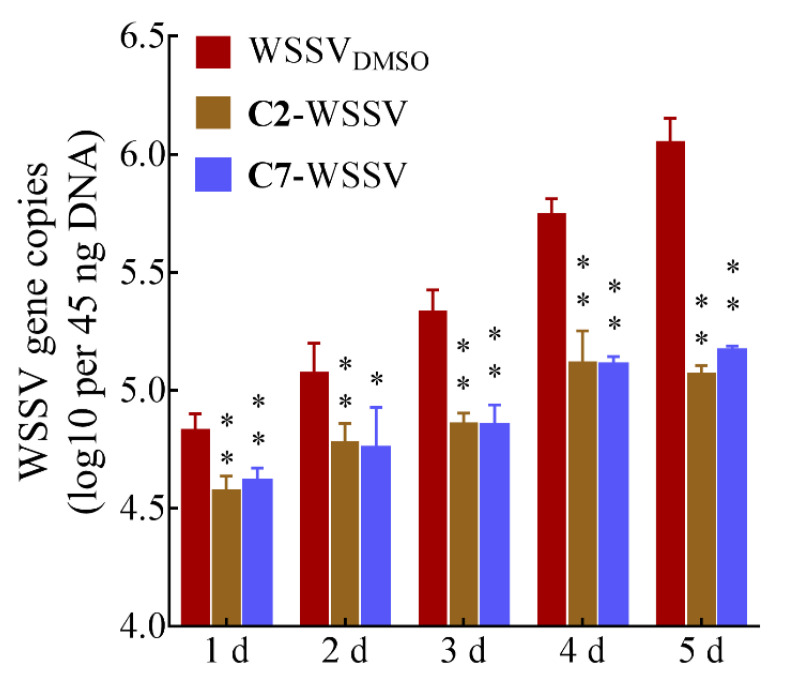
Inhibition of **C2** and **C7** on WSSV horizontal transmission. Each value is represented as the mean ± SD normalized to values for no treatment. The *p* value for each study was determined by Student’s *t* tests. ** *p* < 0.01; * *p* < 0.05.

The nature of the water environment results in a rapid transmission of WSSV within shrimp populations. A typical outbreak starts in the first day or two after the introduction of virus or virus-infected shrimp, followed by a mass mortality of 70–100% in the next five to seven days [43]. This is primarily due to horizontal transmission of the virus [44]. Exploring the compound efficiency against horizontal WSSV transmission is important to break the cycle of epizootic outbreaks and reducing the persistence of the virus. In this study, WSSV replication in naïve recipient larvae was significantly downregulated after **C2** and **C7** exposure at each time point (Figure 7). Taken together with the results of earlier studies [24,33], our findings suggest that antiviral coumarins are suitable for static aquaculture systems and for the culture of shrimp seedlings.

## 3. Materials and Methods

### 3.1. Reagents

All conventional reagents and solvents are purchased directly from commercial companies and no further purification is required. Organic solvents were purchased from Sinopharm chemical reagent Co., Ltd. and purified by distillation and moisture was excluded from the glass apparatus using CaCl_2_ drying tubes. Throughout this study, silica gel H (200–300 mesh; Qingdao Marine Chemical Factory, Qingdao, China) was used for the column chromatography. For thin layer chromatographic (TLC) plates, Silica gel (GF254) (Qingdao Marine Chemical Factory, Qingdao, China) were used for thin layer chromatographic (TLC) analysis, and all of the spots and bands were detected by UV irradiation (254, 365 nm). The intermediates and products were characterized by electrospray ionization mass spectrometry (TripleTOF5600þ; AB SCIEX Pte. Ltd., Boston, MA, USA), ^1^H NMR and ^13^C NMR (Bruker AM500 spectrometer; Bruker Corporation, Berne, Switzerland). The derivatives were dissolved in 100% dimethyl sulfoxide (DMSO; Beyotime Biotechnology, Haimen, China), stored at 4 °C prior to antiviral evaluation, and used within 6 months. For solubility, the stock solutions, at a concentration of 50 mg/mL, were sonicated for approximately 10 min to ensure that the derivatives were fully dissolved and mixed.

### 3.2. Synthesis of Coumarin Derivatives

**Figure 8:**
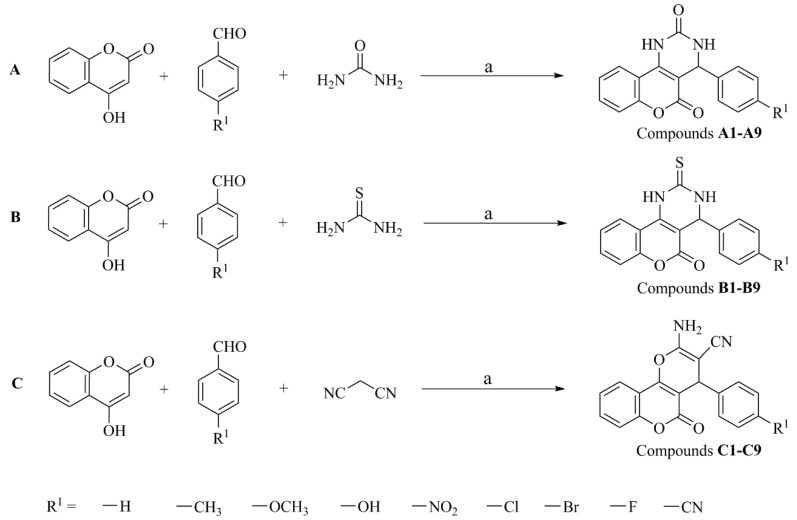
Synthetic route of coumarin derivatives **A1**–**9**, **B1**–**9**, and **C1**–**9**. Reagents and conditions: (a) 0.1 M SLS, H_2_O, 60–65 °C, 5 h.

Twenty-seven coumarin derivatives were synthesized using coumarin as the initial compound (Figure 8). A mixture of 4-hydroxy coumarin (2 mmol), aromatic aldehydes (2 mmol), urea (3 mmol)/sulfourea (3 mmol)/malononitrile (2 mmol), and SLS (10 mol) in water (20 mL) was subjected to ultrasound bath for 0.5 h. Then, the mixture was refluxed at 102 °C for 5 h. Process of the reaction was monitored by TLC. After 5 h, the reaction mixture was cold to room temperature, filtered and washed with hot water. The products were recrystallized from dichloromethane to give the target compounds. By analyzing the data of ^1^H nuclear magnetic resonance (NMR), ^13^C NMR, and electrospray ionization mass spectrometry (ESI-MS) mass-to-charge ratio (*m/z*), coumarins were identified. The related data of **A1**–**9** were described as followed.

*4-phenyl-3,4-dihydro-2H-chromeno[4,3-d]pyrimidine-2,5(1H)-dione (**A1**)*: yield 74.4%, white powder. ^13^C NMR (126 MHz, DMSO) δ 165.41, 164.81, 152.22, 140.02, 131.84, 128.03, 126.69, 125.52, 123.91, 123.69, 118.02, 115.92, 104.08, 35.98. ^1^H NMR (500 MHz, DMSO) δ 7.93–7.91, 7.63–7.59, 7.3–7.32, 7.26–7.23, 7.18–7.16, 6.39. HRMS (ESI) calcd for C_17_H_13_N_2_O_3_ [M+H]^+^ 293.0926, found 293.0921.

*4-(p-tolyl)-3,4-dihydro-2H-chromeno[4,3-d]pyrimidine-2,5(1H)-dione (**A2**)*: yield 70.0%, white powder. ^13^C NMR (126 MHz, DMSO) δ 165.19, 164.81, 152.18, 136.70, 134.42, 131.86, 128.66, 126.61, 123.88, 123.73, 117.92, 115.94, 104.24, 35.62, 20.51. ^1^H NMR (500 MHz, DMSO-*d*_6_) δ 7.92 (dd, *J* = 7.9, 1.7 Hz, 2H), 7.61 (ddd, *J* = 8.7, 7.2, 1.7 Hz, 2H), 7.38 (d, *J* = 8.3 Hz, 2H), 7.36–7.31 (m, 2H), 7.05 (s, 4H), 6.33 (s, 1H), 2.26 (s, 3H). HRMS (ESI) calcd for C_18_H_15_N_2_O_3_ [M+H]^+^ 307.1083, found 307.1089.

*4-(4-methoxyphenyl)-3,4-dihydro-2H-chromeno[4,3-d]pyrimidine-2,5(1H)-dione (**A3**)*: yield 68.6%, white powder. ^13^C NMR (126 MHz, DMSO) δ 167.39, 164.58, 156.82, 152.45, 133.85, 130.91, 127.61, 124.06, 122.90, 119.79, 115.46, 113.14, 103.72, 54.88, 35.35. ^1^H NMR (500 MHz, DMSO-*d*_6_) δ 7.83 (dd, *J* = 7.9, 1.6 Hz, 2H), 7.54–7.48 (m, 2H), 7.29–7.18 (m, 5H), 7.09 (s, 1H), 7.03–6.98 (m, 3H), 6.74 (d, *J* = 8.7 Hz, 2H), 6.22 (s, 1H), 3.69 (s, 3H). HRMS (ESI) calcd for C_18_H_15_N_2_O_4_ [M+H]^+^ 323.1032, found 323.1030.

*4-(4-hydroxyphenyl)-3,4-dihydro-2H-chromeno[4,3-d]pyrimidine-2,5(1H)-dione (**A4**)*: yield 65.3%, white powder. ^13^C NMR (126 MHz, DMSO) δ 164.82, 155.33, 152.13, 131.92, 129.31, 127.69, 123.84, 123.80, 117.74, 115.97, 114.95, 104.54, 35.20. ^1^H NMR (500 MHz, DMSO-*d*_6_) δ 7.93 (dd, *J* = 7.9, 1.7 Hz, 2H), 7.66–7.57 (m, 2H), 7.43–7.31 (m, 4H), 6.96 (d, *J* = 8.3 Hz, 2H), 6.68–6.63 (m, 2H), 6.27 (s, 1H). HRMS (ESI) calcd for C_17_H_13_N_2_O_4_ [M+H]^+^ 309.0875, found 309.0878.

*4-(4-fluorophenyl)-3,4-dihydro-2H-chromeno[4,3-d]pyrimidine-2,5(1H)-dione (**A5**)*: yield 80.6%, white powder. ^13^C NMR (126 MHz, DMSO) δ 165.65, 164.69, 152.28, 136.29, 131.81, 128.58, 128.52, 123.95, 123.64, 118.19, 115.91, 114.70, 114.68, 114.53, 104.05, 35.51. ^1^H NMR (500 MHz, DMSO-*d*_6_) δ 7.92 (dd, *J* = 7.9, 1.6 Hz, 2H), 7.60 (td, *J* = 7.8, 7.3, 1.6 Hz, 2H), 7.37 (d, *J* = 8.3 Hz, 2H), 7.33 (t, *J* = 7.5 Hz, 2H), 7.19 (dd, *J* = 8.4, 5.4 Hz, 2H), 7.05 (t, *J* = 8.8 Hz, 2H), 6.34 (s, 1H). HRMS (ESI) calcd for C_17_H_12_ N_2_O_3_F [M+H]^+^ 311.0832, found 311.0829.

*4-(4-chlorophenyl)-3,4-dihydro-2H-chromeno[4,3-d]pyrimidine-2,5(1H)-dione (**A6**)*: yield 60.7%, white powder. ^13^C NMR (126 MHz, DMSO) δ 165.84, 164.61, 152.29, 139.69, 131.76, 129.95, 128.68, 127.86, 123.95, 123.57, 118.29, 115.87, 103.75, 35.71. ^1^H NMR (500 MHz, DMSO-*d*_6_) δ 7.91 (dd, *J* = 8.0, 1.7 Hz, 2H), 7.60 (ddd, *J* = 8.7, 7.3, 1.7 Hz, 2H), 7.39–7.30 (m, 4H), 7.30–7.25 (m, 2H), 7.21–7.16 (m, 2H), 6.33 (s, 1H). HRMS (ESI) calcd for C_17_H_12_ClN_2_O_3_ [M+H]^+^ 327.0536, found 327.0537.

*4-(4-bromophenyl)-3,4-dihydro-2H-chromeno[4,3-d]pyrimidine-2,5(1H)-dione (**A7**)*: yield 66.9%, white powder. ^13^C NMR (126 MHz, DMSO) δ 166.27, 164.55, 152.35, 140.59, 131.61, 130.73, 129.10, 124.00, 123.44, 118.63, 118.26, 115.80, 103.55, 35.80. ^1^H NMR (500 MHz, DMSO-*d*_6_) δ 7.88 (dd, *J* = 7.9, 1.6 Hz, 2H), 7.57 (ddd, *J* = 8.6, 7.3, 1.7 Hz, 2H), 7.39 (dd, *J* = 9.3, 3.1 Hz, 2H), 7.37–7.27 (m, 4H), 7.10 (d, *J* = 8.2 Hz, 2H), 6.28 (s, 1H). HRMS (ESI) calcd for C_17_H_12_BrN_2_O_3_ [M+H]^+^ 371.0031, found 371.0033.

*4-(4-nitrophenyl)-3,4-dihydro-2H-chromeno[4,3-d]pyrimidine-2,5(1H)-dione (**A8**)*: yield 82.1%, yellow powder. ^13^C NMR (126 MHz, DMSO) δ 167.92, 164.33, 152.56, 151.48, 145.29, 131.19, 127.85, 124.17, 123.09, 123.01, 119.68, 115.56, 102.74, 36.75. ^1^H NMR (500 MHz, DMSO-*d*_6_) δ 8.08 (d, *J* = 8.8 Hz, 2H), 7.83 (dd, *J* = 7.9, 1.5 Hz, 2H), 7.58–7.51 (m, 2H), 7.38 (d, *J* = 8.5 Hz, 2H), 7.30 (d, *J* = 8.2 Hz, 2H), 7.25 (t, *J* = 7.5 Hz, 2H), 7.20 (s, 1H), 7.09 (s, 1H), 6.99 (s, 1H), 6.37 (s, 1H). HRMS (ESI) calcd for C_17_H_12_N_3_O_5_ [M+H]^+^ 338.0777, found 338.0783.

*4-(2,5-dioxo-1,3,4,5-tetrahydro-2H-chromeno[4,3-d]pyrimidin-4-yl)benzonitrile (**A9**)*: yield 83.6%, white powder. ^13^C NMR (126 MHz, DMSO) δ 166.18, 164.50, 152.39, 147.46, 131.94, 131.80, 127.93, 124.02, 123.56, 119.16, 118.39, 115.90, 108.20, 103.31, 36.68. ^1^H NMR (500 MHz, DMSO-*d*_6_) δ 7.91 (dd, *J* = 7.9, 1.7 Hz, 2H), 7.70 (d, *J* = 8.3 Hz, 2H), 7.65–7.57 (m, 2H), 7.35 (dt, *J* = 25.5, 7.5 Hz, 6H), 6.40 (s, 1H). HRMS (ESI) calcd for C_18_H_12_N_3_O_3_ [M+H]^+^ 318.0879, found 318.0871.

*4-phenyl-2-thioxo-1,2,3,4-tetrahydro-5H-chromeno[4,3-d]pyrimidin-5-one (**B1**)*: yield 69.2%, white powder. ^13^C NMR (126 MHz, DMSO) δ 165.30, 164.82, 152.21, 139.91, 131.89, 128.06, 126.70, 125.56, 123.90, 123.74, 117.93, 115.95, 104.12, 35.97. ^1^H NMR (500 MHz, DMSO) δ 7.94–7.92, 7.64–7.60, 7.40–7.33, 7.26–7.23, 7.18–7.15, 6.39. HRMS (ESI) calcd for C_17_H_13_SN_2_O_2_ [M+H]^+^ 309.0698, found 309.0693.

*2-thioxo-4-(p-tolyl)-1,2,3,4-tetrahydro-5H-chromeno[4,3-d]pyrimidin-5-one (**B2**)*: yield 65.6%, white powder. ^13^C NMR (126 MHz, DMSO) δ 165.05, 164.82, 152.16, 136.56, 134.47, 131.90, 128.67, 126.60, 123.86, 123.76, 117.81, 115.95, 104.28, 35.61, 20.51. ^1^H NMR (500 MHz, DMSO-*d*_6_) δ 7.93 (d, *J* = 7.9, 1.7 Hz, 2H), 7.70–7.54 (m, 2H), 7.45–7.29 (m, 4H), 7.05 (s, 4H), 6.34 (s, 1H), 2.26 (s, 3H). HRMS (ESI) calcd for C_18_H_15_SN_2_O_2_ [M+H]^+^ 323.0854, found 323.0844.

*4-(4-methoxyphenyl)-2-thioxo-1,2,3,4-tetrahydro-5H-chromeno[4,3-d]pyrimidin-5-one (**B3**)*: yield 64.4%, white powder. ^13^C NMR (126 MHz, DMSO) δ 165.11, 164.78, 157.30, 152.17, 131.85, 131.46, 127.75, 123.86, 123.72, 117.91, 115.93, 113.48, 104.37, 54.95, 35.25. ^1^H NMR (500 MHz, DMSO-*d*_6_) δ 7.92 (dd, *J* = 7.9, 1.5 Hz, 2H), 7.70–7.56 (m, 2H), 7.46–7.29 (m, 4H), 7.07 (d, *J* = 8.3 Hz, 2H), 6.81 (d, *J* = 8.4 Hz, 2H), 6.31 (s, 1H), 3.72 (s, 3H). HRMS (ESI) calcd for C_18_H_15_SN_2_O_3_ [M+H]^+^ 339.0803, found 339.0808.

*4-(4-hydroxyphenyl)-2-thioxo-1,2,3,4-tetrahydro-5H-chromeno[4,3-d]pyrimidin-5-one (**B4**)*: yield 62.4%, white powder. ^13^C NMR (126 MHz, DMSO) δ 164.82, 164.72, 155.35, 152.11, 131.94, 129.20, 127.68, 123.82, 117.66, 115.97, 114.96, 104.56, 35.20. ^1^H NMR (500 MHz, DMSO-*d*_6_) δ 7.93 (dd, *J* = 8.0, 1.7 Hz, 2H), 7.74–7.54 (m, 2H), 7.51–7.25 (m, 4H), 6.97 (d, *J* = 8.2 Hz, 2H), 6.66 (d, *J* = 8.2 Hz, 2H), 6.28 (s, 1H). HRMS (ESI) calcd for C_17_H_13_SN_2_O_3_ [M+H]^+^ 325.0647, found 325.0638.

*4-(4-fluorophenyl)-2-thioxo-1,2,3,4-tetrahydro-5H-chromeno[4,3-d]pyrimidin-5-one (**B5**)*: yield 61.7%, white powder. ^13^C NMR (126 MHz, DMSO) δ 165.61, 164.68, 161.40, 159.49, 152.26, 136.27, 131.80, 128.57, 128.51, 123.93, 123.63, 118.17, 115.90, 114.68, 114.52, 104.05, 35.50. ^1^H NMR (500 MHz, DMSO) δ 7.92–7.91 (m), 7.62–7.59 (m), 7.38–7.31 (m), 7.20–7.18 (m), 7.06–7.03 (t), 6.33 (s, 1H). HRMS (ESI) calcd for C_17_H_12_SN_2_O_2_F [M+H]^+^ 327.0604, found 327.0612.

*4-(4-chlorophenyl)-2-thioxo-1,2,3,4-tetrahydro-5H-chromeno[4,3-d]pyrimidin-5-one (**B6**)*: yield 65.6%, white powder. ^13^C NMR (126 MHz, DMSO) δ 165.74, 164.61, 152.28, 139.60, 131.79, 129.98, 128.69, 127.87, 123.94, 123.60, 118.21, 115.89, 103.78, 35.71. ^1^H NMR (500 MHz, DMSO) δ 7.92–7.90, 7.62–7.58, 7.38–7.27, 7.19–7.18, 6.33 (s, 1H). HRMS (ESI) calcd for C_17_H_12_SN_2_O_2_Cl [M+H]^+^ 343.0308, found 343.0303.

*4-(4-bromophenyl)-2-thioxo-1,2,3,4-tetrahydro-5H-chromeno[4,3-d]pyrimidin-5-one (**B7**)*: yield 66.1%, white powder. ^13^C NMR (126 MHz, DMSO) δ 165.70, 164.62, 152.28, 140.05, 131.82, 130.80, 129.12, 123.94, 123.63, 118.44, 118.17, 115.90, 103.74, 35.78. ^1^H NMR (500 MHz, DMSO) δ 7.93–7.91, 7.62–7.59, 7.42–7.32, 7.14–7.13, 6.32. HRMS (ESI) calcd for C_17_H_12_SN_2_O_2_Br [M+H]^+^ 386.9803, found 386.9798.

*4-(4-nitrophenyl)-2-thioxo-1,2,3,4-tetrahydro-5H-chromeno[4,3-d]pyrimidin-5-one (**B8**)*: yield 74.6%, yellow powder. ^13^C NMR (126 MHz, DMSO) δ 166.60, 164.38, 152.44, 150.31, 145.51, 131.68, 128.04, 124.06, 123.44, 123.19, 118.69, 115.83, 103.23, 36.73. ^1^H NMR (500 MHz, DMSO-*d*_6_) δ 8.10 (d, *J* = 8.8 Hz, 2H), 7.89 (dd, *J* = 7.9, 1.6 Hz, 2H), 7.68–7.55 (m, 2H), 7.43 (d, *J* = 8.5 Hz, 2H), 7.35 (d, *J* = 8.3 Hz, 2H), 7.31 (t, *J* = 7.6 Hz, 2H), 6.41 (s, 1H). HRMS (ESI) calcd for C_17_H_12_SN_3_O_4_ [M + H]^+^ 354.0549, found 354.0556.

*4-(5-oxo-2-thioxo-1,3,4,5-tetrahydro-2H-chromeno[4,3-d]pyrimidin-4-yl)benzonitrile (**B9**)*: yield 63.1%, white powder. ^13^C NMR (126 MHz, DMSO) δ 166.61, 164.43, 152.41, 147.87, 131.87, 131.60, 127.85, 124.03, 123.38, 119.17, 118.73, 115.78, 108.03, 103.11, 36.67. ^1^H NMR (500 MHz, DMSO) δ 8.09, 7.88–7.86, 7.68–7.66, 7.59–7.56, 7.35–7.28, 6.37 (s, 1H). HRMS (ESI) calcd for C_18_H_12_SN_3_O_2_ [M+H]^+^ 334.0650, found 334.0651.

### 3.3. Shrimp Experiments and Acute Toxicity Assay

Shrimp (*L. vannamei*) larvae (total length: 0.5 ± 0.04 cm, body weight: 4.2 ± 0.4 mg, mean ± SD) were provided by Zhejiang Mariculture Research Institute Qingjiang Station (Wenzhou, China). The larvae were maintained in a culture pond at 28 °C, fed four times a day with commercial feed pellets, and collected randomly for PCR testing to verify the WSSV-free status prior to the trial.

Acute toxicity of the coumarin derivatives was investigated by exposing larvae to a range of compound concentrations, from 0.1 mg/L to 100 mg/L of coumarin derivatives, where the dose setting was based on the data from preliminary tests in our laboratory. A DMSO-control (2%, *v/v*, the highest percentage of DMSO in treatments) served as the control. During the experiment, every 20 larvae were moved into six-well plates filled with 6 mL/per well freshly prepared test solutions for a 72-h incubation at 28 °C with a natural photoperiod. The larvae were observed at regular intervals (every 6 h); optical microscopy was used to ascertain shrimp death, defined as no movement or response to gentle prodding. To avoid deterioration of water quality, dead shrimps were immediately removed at the point of observation. Three replicate experiments were performed.

### 3.4. WSSV Propagation

Stock concentrations of WSSV were obtained from the aquaculture laboratory of the Zhejiang Mariculture Research Institute. For in vivo studies, due to the inability to inject them, the larvae were immersed in WSSV diluent (final viral titer of 1.6 × 10^5^ copies/µL). In order to simulate natural infection pathways, according to previous studies on the major route of WSSV natural infection [45,46,47], healthy larvae in this study ingested the tissue homogenate of WSSV-infected sick or dead shrimp to amplify the virus, given the cannibalistic nature of shrimp.

### 3.5. In Vivo Inhibition

For pre-incubation, shrimp larvae were randomly selected, separated into six-well plates containing ultraviolet light-sterilized aquacultural water, and infected with WSSV (final viral titer of 1.6 × 10^5^ copies/µL) in the presence of coumarins for 72 h. Mock negative controls (1% DMSO) were also used. The homogenate of each well containing sick or dead shrimp was subjected to low-speed centrifugation (2000× *g*). The viral load was quantified by determining the copy number of the viral genome in samples by quantitative reverse transcription polymerase chain reaction (RT-qPCR) assay, as previously described [32].

For preincubation with immersion, WSSV was preincubated with **C2/C7** to a final viral titer of 1.6 × 10^5^ copies/µL and up to 10 mg/L coumarin compound or vehicle control in the challenge wells. After 1, 2, and 4 h of incubation, shrimp larvae were added to the challenge well until 72-h post-infection. The surviving shrimp were humanely euthanized with tricaine methane sulfonate (MS-222) at a final concentration of 150 mg/L, followed by quantification of the copy number of the viral genome.

For pre-treatment, **C2/C7** (final concentration of 10 mg/L) was applied to shrimp larvae for 1, 4, and 8 h, followed by addition of WSSV (while the coumarin compounds were simultaneously removed) at a final viral titer of 1.6 × 10^5^ copies/µL. The surviving shrimps were euthanized with MS-222 and frozen at −80 °C until being processed. The copy number of viral genome in samples was quantified by RT-qPCR.

For continuous immersion with cultural medium exchange, the culture water in each well was replaced by **C2/C7** or DMSO solution after WSSV infection (final viral titer of 1.6 × 10^5^ copies/µL) for 24 h. Every 24 h, the shrimp were transformed into a new challenge well with fresh water and a fresh dose of **C2/C7** or DMSO (0.2‰) for an additional 5 days. The survival shrimps were euthanized with MS-222 and frozen at −80 °C until processing. The survival of shrimp larvae and the copy number of viral genome in samples were described as above.

### 3.6. Stability Assay

Aquaculture water was obtained from culture ponds of the Zhejiang Mariculture Research Institute Qingjiang Station. **C2/C7,** with a final concentration of 10 mg/L, was added to aquacultural water samples in the presence of natural light for up to 4 days, at 28 °C, and finally preincubated with WSSV (final viral titer of 1.6 × 10^5^ copies/µL) to infect shrimp larvae, followed by quantification of the copy number of the viral genome at 72 hpi, and the survival rate of the shrimp was evaluated.

### 3.7. Horizontal Transmission

Shrimp larvae were infected with 1.6 × 10^5^ copies/µL WSSV or aquacultural water only. At 24 hpi, a total of the 12 donor shrimps were placed into each challenge beaker (100 mL of water and a continuous air supply), and C2 (10 mg/L final concentration) or DMSO (0.2‰ final concentration) was added to each challenge beaker. Every 24 h, five donor and fifteen recipient shrimp were netted into a new challenge beaker with fresh water and a fresh dose of **C2/C7** (10 mg/L) or DMSO (0.2‰). After 72 h of cohabitation, the surviving recipient shrimps were euthanized with MS-222 and frozen at −80 °C. In order to distinguish the donor and recipient samples in this test, we chose the larger shrimp as the recipients (total length of 1.1 ± 0.2 cm, body weight of 6.9 ± 0.5 mg, mean values ± SD) and placed a sieve in the challenge beaker to separate the donor and recipient shrimps.

### 3.8. Statistical Analyses

Compound response curves were represented by a logistic sigmoidal function with a maximal effect level (Amax) and Hill coefficient representing the sigmoidal transition (Origin 8.1; http://www.originlab.com, accessed on 25 March 2021). Statistical analyses were performed using SPSS (version 18.0) statistical software (SPSS Inc., Chicago, IL, USA). The LC_50_ and EC_50_ values with 95% CIs were calculated using probit analysis. Virus titers were log10-transformed prior to statistical analyses. Unpaired Student’s *t*-tests were used to compare coumarin-treated samples and negative-control DMSO samples for determining significance and data are presented as mean values ± SD.

## 4. Conclusions

In this study, a series of coumarin derivatives were designed and synthesized, and their antiviral efficacy against WSSV infection in *L. vannamei* larvae was evaluated. The SAR study identified that toxicity and antiviral activity is represented by the **R^1^** substituent: electron-withdrawing groups reduced toxicity, and electron-donating groups markedly increased antiviral activity. After analysis of MAR and EC_50_ values, two coumarin derivatives, **C2** (2-amino-5-oxo-4-(*p*-tolyl)-4H,5H-pyrano[3,2-c]chromene-3-carbonitrile), and **C7** (2-amino-4-(4-chlorophenyl)-5-oxo-4H,5H-pyrano[3,2-c]chromene-3-carbonitrile), demonstrated the most promising antiviral effects. These results revealed that **C2** and **C7** strongly protected larvae from WSSV infection, are stable in the aquaculture environment, and are useful as therapeutic agents against horizontal transmission of the virus.

## Figures and Tables

**Table 1:** Medium lethal concentration (LC_50_; mg/L), median effective concentration (EC_50_; mg/L) and maximum antiviral response (MAR; %) of coumarin derivatives.

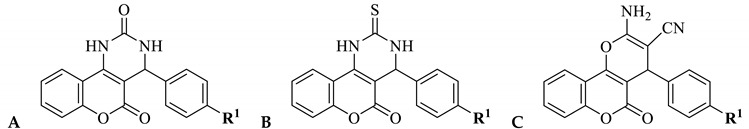						
Coumarin	Substituent Group	LC_50_ (mg/L; 95% CI)			EC_50_ (mg/L; 95% CI)	MAR (%)
	R^1^	24 h	48 h	72 h		
**A1**	-H	95.538 (85.361~110.004)	71.362 (64.016~79.198)	32.547 (28.664~36.084)	21.547 (18.272~28.977)	45.420 ± 5.349
**A2**	-CH_3_	15.015 (13.025~17.267)	14.260 (11.996~16.866)	10.016 (7.943~12.593)	05.037 (4.076~7.593)	48.126 ± 12.170
**A3**	-OCH_3_	26.878 (24.736~29.161)	24.493 (21.367~27.876)	21.113 (19.113~23.177)	14.906 (13.804~16.467)	29.056 ± 6.706
**A4**	-OH	NONE (50)	NONE (50)	NONE (50)	21.735 (18.272~27.292)	06.674 ± 1.126
**A5**	-F	16.632 (14.445~19.142)	11.745 (9.552~14.182)	10.058 (9.341~10.749)	08.416 (7.137~11.317)	46.729 ± 8.371
**A6**	-Cl	02.584 (2.375~2.801)	02.481 (2.265~2.708)	02.346 (2.102~2.612)	02.019 (1.698~2.752)	37.829 ± 8.606
**A7**	-Br	14.138 (12.682~15.721)	05.683 (3.835~7.947)	03.367 (3.236~3.504)	NONE	32.763 ± 6.196
**A8**	-NO_2_	8.762 (7.566~10.149)	03.427 (2.382~4.668)	01.614 (0.876~2.076)	02.524 (2.102~3.686)	33.041 ± 6.184
**A9**	-CN	19.827 (16.048~25.013)	17.954 (14.245~22.999)	07.325 (6.031~8.841)	NONE	43.976 ± 6.775
**B1**	-H	15.009 (13.077~17.305)	12.753 (11.412~14.272)	10.533 (9.939~11.163)	NONE	61.107 ± 10.144
**B2**	-CH_3_	23.856 (21.389~26.646)	18.584 (17.058~20.239)	14.678 (13.474~16.144)	NONE	48.247 ± 10.554
**B3**	-OCH_3_	66.188 (56.660~79.101)	64.053 (54.821~76.464)	41.706 (39.817~43.740)	25.633 (19.809~44.856)	38.060 ± 5.105
**B4**	-OH	NONE (50)	NONE (50)	NONE (50)	22.620 (20.063~26.163)	09.678 ± 1.090
**B5**	-F	16.552 (14.508~18.865)	08.869 (7.444~10.576)	08.397 (6.852~10.280)	04.628 (4.2003~5.248)	43.210 ± 9.573
**B6**	-Cl	08.847 (7.315~10.641)	06.630 (4.493~9.424)	04.501 (4.221~4.796)	NONE	43.073 ± 7.090
**B7**	-Br	10.020 (8.540~11.733)	04.812 (4.547~5.065)	04.465 (4.192~4.752)	NONE	53.699 ± 10.979
**B8**	-NO_2_	13.551 (12.055~15.176)	07.476 (6.653~8.381)	03.057 (2.553~3.598)	02.563 (1.981~4.486)	32.411 ± 4.334
**B9**	-CN	37.071 (32.610~43.015)	31.491 (28.115~35.385)	09.629 (7.268~12.684)	NONE	41.424 ± 8.477
**C1**	-H	18.425 (12.898~28.819)	16.639 (11.315~27.121)	09.176 (7.179~11.753)	02.903 (2.635~3.251)	10.170 ± 2.281
**C2**	-CH_3_	NONE (16)	NONE (16)	NONE (16)	05.993 (5.327~6.851)	01.231 ± 0.226
**C3**	-OCH_3_	22.324 (20.942~23.748)	17.328 (15.718~19.118)	17.328 (15.718~19.118)	14.816 (13.661~16.424)	29.395 ± 7.170
**C4**	-OH	NONE	NONE	NONE	61.167 (55.018~70.481)	09.678 ± 1.090
**C5**	-F	NONE (16)	NONE (16)	NONE (16)	08.824 (7.921~10.023)	04.122 ± 0.502
**C6**	-Cl	NONE	44.227 (31.822~92.192)	17.679 (14.965~20.890)	08.535 (7.881~9.499)	43.073 ± 7.090
**C7**	-Br	NONE (16)	NONE (16)	NONE (16)	05.727 (5.069~6.577)	03.154 ± 1.157
**C8**	-NO_2_	NONE (16)	NONE (16)	NONE (16)	06.603 (5.410~8.396)	03.262 ± 0.218
**C9**	-CN	NONE	NONE	NONE	60.472 (53.129~72.065)	41.424 ± 8.477

CI represents confidence interval. Blue font “NONE” represents that no valid data were tested for statistical analysis by SPSS 18.0. Red data with asterisks represent the lowest precipitation concentration of coumarins within the test concentration range of 0.4~100 mg/L.

## Data Availability

Data are contained within the article.

## References

[1] Li C., Wang S., He J. (2019). The two NF-κB pathways regulating bacterial and WSSV infection of shrimp. Front. Immunol..

[2] Thitamadee S., Prachumwat A., Srisala J., Jaroenlak P., Salachan P.V., Sritunyalucksana K., Flegel T.W., Itsathitphaisarn O. (2016). Review of current disease threats for cultivated penaeid shrimp in Asia. Aquaculture.

[3] Lightner D.V., Redman R.M., Pantoja C.R., Tang K.F.J., Noble B.L., Schofield P., Mohney L.I., Nunan L.M., Navarro S.A. (2012). Historic emergence, impact and current status of shrimp pathogens in the Americas. J. Invert. Pathol..

[4] Peeler E.J. (2012). Costs and benefits of freedom from shrimp diseases in the European Union. J. Invert. Pathol..

[5] 46th ESAO Congress 3–7 September 2019 Hannover, Germany. https://journals.sagepub.com/doi/abs/10.1177/0391398819860985?journalCode=jaoa.

[6] Inouye K., Miwa S., Oseko N., Nakano H., Hiraoka M. (1994). Mass mortalities of cultured kuruma shrimp *Penaeus japonicus* in Japan in 1993: Histopathological study. Fish Pathol..

[7] Stentiford G.D., Lightner D.V. (2011). Cases of white spot disease (WSD) in European shrimp farms. Aquaculture.

[8] Kathy F.J., Tang M., Groumellec L., Lightner D.V. (2013). Novel, closely related, white spot syndrome virus (WSSV) genotypes from Madagascar, Mozambique and the Kingdom of Saudi Arabia. Dis. Aqua. Organ..

[9] Oakey H.J., Smith C.S. (2018). Complete genome sequence of a white spot syndrome virus associated with a disease incursion in Australia. Aquaculture.

[10] Mayo M.A. (2002). A summary of taxonomic changes recently approved by ICTV. Arch. Virol..

[11] Lo C.F., Hsu H.C., Tsai M.F., Ho C.H., Peng S.E., Kou G.H., Lightner D.V. (1999). Specific genomic DNA fragment analysis of different geographical clinical samples of shrimp white spot syndrome virus. Dis. Aqua. Organ..

[12] Lightner D.V. (2012). Global transboundry disease politics: The OIE perspective. J. Invert. Pathol..

[13] Kannan S., Kolandaivel P. (2017). Antiviral potential of natural compounds against influenza virus hemagglutinin. Comp. Biol. Chem..

[14] Hu Y., Liu L., Li B.Y., Shen Y.F., Wang G.X., Zhu B. (2019). Synthesis of arctigenin derivatives against infectious hematopoietic necrosis virus. Eur. J. Med. Chem..

[15] Mathew D., Hsu W.L. (2018). Antiviral potential of curcumin. J. Funct. Food..

[16] Mishra S., Pandey A., Manvati S. (2020). Coumarin: An emerging antiviral agent. Heliyon.

[17] Chen W.C., Liu L., Shen Y.F., Hu Y., Ling F., Wang G.X., Zhu B. (2018). A new coumarin derivative plays a role in rhabdoviral clearance by interfering glycoprotein function during the early stage of viral infection. Cell. Signal..

[18] Liu L., Shen Y.F., Hu Y., Lu J.F. (2018). Antiviral effect of 7-(4-benzimidazole-butoxy)-coumarin on rhabdoviral clearance via Nrf2 activation regulated by PKC α/β phosphorylation. Fish Shellfish Immunol..

[19] Liu L., Song D.W., Liu G.L., Shan L.P., Qiu T.X., Chen J. (2020). Hydroxycoumarin efficiently inhibits spring viraemia of carp virus infection in vitro and in vivo. Zool. Res..

[20] Li G., Gao Q., Yuan S., Wang L., Altrneyer R., Lan K., Yin F.F., Zou G. (2017). Characterization of three small molecule inhibitors of enterovirus 71 identified from screening of a library of natural products. Antivir. Res..

[21] Liu L., Hu Y., Shen Y.F., Wang G.X., Zhu B. (2017). Evaluation on antiviral activity of coumarin derivatives against spring viraemia of carp virus in epithelioma papulosum cyprini cells. Antivir. Res..

[22] Song D.W., Liu L., Shan L.P., Qiu T.X., Chen J., Chen J.P. (2020). Therapeutic potential of phenylpropanoid-based small molecules as anti-SVCV agents in aquaculture. Aquaculture.

[23] Song D.W., Liu L., Shan L.P., Qiu T.X., Chen J., Chen J.P. (2020). Rhabdoviral clearance effect of a phenylpropanoid medicine against spring viremia of carp virus infection in vitro and in vivo. Aquaculture.

[24] Qiu T.X., Song D.W., Shan L.P., Liu G.L., Liu L. (2020). Potential prospect of a therapeutic agent against spring viraemia of carp virus in aquaculture. Aquaculture.

[25] Rizan N., Yew C.Y., Niknam M.R., Krishnasamy J., Bhassu S., Hong G.Z., Devadas S., Din M.S.M., Tajuddin H.A., Othman R.Y. (2018). Electronic properties of synthetic shrimp pathogens-derived DNA schottky diodes. Sci. Rep..

[26] Phuthaworn C., Nguyen N.H., Quinn J., Knibb W. (2016). Moderate heritability of hepatopancreatic parvovirus titre suggests a new option for selection against viral diseases in banana shrimp (*Fenneropenaeus merguiensis*) and other aquaculture species. Genet. Sel. Evol..

[27] Bachère E., Gueguen Y., Gonzalez M., de Lorgeril J., Garnier J., Romestand B. (2004). Insights into the anti-microbial defense of marine invertebrates: The penaeid shrimps and the oyster *Crassostrea gigas*. Immunol. Rev..

[28] Sanchez-Paz A. (2010). White spot syndrome virus: An overview on an emergent concern. Vet. Res..

[29] Citarasu T., Sivaram V., Immanuel G., Rout N., Murugan V. (2006). Influence of selected Indian immunostimulant herbs against white spot syndrome virus (WSSV) infection in black tiger shrimp, *Penaeus monodon* with reference to haematological, biochemical and immunological changes. Fish Shellfish Immunol..

[30] Rameshthangam P., Ramasamy P. (2007). Antiviral activity of bis(2-methylheptyl)phthalate isolated from *Pongamia pinnata* leaves against white spot syndrome virus of *Penaeus monodon* fabricius. Virus Res..

[31] Chen C., Shen Y.F., Hu Y., Liu L., Chen W.C., Wang G.X., Zhu B. (2018). Highly efficient inhibition of spring viraemia of carp virus replication in vitro mediated by bavachin, a major constituent of *Psoralea corlifonia* Lynn. Virus Res..

[32] Huang A.G., Tan X.P., Cui H.B., Qi X.Z., Zhu B., Wang G.X. (2020). Antiviral activity of geniposidic acid against white spot syndrome virus replication in red swamp crayfish *Procambarus clarkia*. Aquaculture.

[33] Liu L., Qiu T.X., Song D.W., Shan L.P., Chen J. (2020). Inhibition of a novel coumarin on an aquatic rhabdovirus by targeting the early stage of viral infection demonstrates potential application in aquaculture. Antivir. Res..

[34] Wang H., Chen Y., Ru G., Xu Y., Lu L. (2018). EGCG: Potential application as a protective agent against grass carp reovirus in aquaculture. J. Fish Dis..

[35] Shen Y.F., Hu Y., Zhang Z., Liu L., Chen C., Tu X., Wang G.X., Zhu B. (2019). Saikosaponin D efficiently inhibits SVCV infection in vitro and in vivo. Aquaculture.

[36] Bourinbaiar A.S., Tan X., Nagorny R. (1993). Effect of the oral anticoagulant, warfarin, on HIV-1 replication and spread. AIDS.

[37] Stubbs M.T., Bode W. (1993). A player of many parts: The spotlight falls on thrombins structure. Thromb. Res..

[38] Sancho R., Marquez N., Gomez-Gonzalo M., Calzado M.A., Bettoni G., Coiras M.T., Alcami J., Lopez-Cabrera M., Appendino G., Munoz E. (2004). Imperatorin inhibits HIV-1 replication through an Sp1-dependent pathway. J. Biol. Chem..

[39] Neyts J., Clercq E.D., Singha R., Chang Y.H., Das A.R., Chakraborty S.K., Hong S.C., Tsay S.C., Hsu M.H., Hwu J.R. (2009). Structure-activity relationship of new anti-hepatitis C virus agents: Heterobicycle-coumarin conjugates. J. Med. Chem..

[40] Balmer B.F., Powers R.L., Zhang T.H., Lee J., Vigant F., Lee B., Jung M.E., Purcell M.K., Snekvik K., Aguilar H.C. (2017). Inhibition of an aquatic rhabdovirus demonstrates promise of a broad-spectrum antiviral for use in aquaculture. J. Virol..

[41] Ahmad I., Rani A.M.B., Verma A.K., Maqsood M. (2017). Biofloc technology: An emerging avenue in aquatic animal healthcare and nutrition. Aquac. Int..

[42] Moss S.M., Moss D.R., Arce S.M., Lightner D.V., Lotz J.M. (2012). The role of selective breeding and biosecurity in the prevention of disease in penaeid shrimp aquaculture. J. Invert. Pathol..

[43] Chou H.Y., Huang C.Y., Wang C.H., Chiang H.C., Lo C.F. (1995). Pathogenicity of a baculovirus infection causing white spot syndrome in cultured penaeid shrimp in Taiwan. Dis. Aqua. Organ..

[44] Rajendran K.V., Vijayan K.K., Santiago T.C., Rajan J. (2005). White spot syndrome virus (WSSV) infection in tiger shrimp Penaeus monodon: A non-lethal histopathological rapid diagnostic method using paraffin and frozen sections. Aquac. Int..

[45] Wu J.L., Namikoshi A., Nishizawa T., Mushiake K., Teruya K., Muroga K. (2001). Effects of shrimp density on transmission of penaeid acute viremia in *Penaeus japonicus* by cannibalism and the waterborne route. Dis. Aqua. Organ..

[46] Soto M.A., Lotz J.M. (2001). Epidemiological parameters of white spot syndrome virus infections in *Litopenaeus vannamei* and *L. setiferus*. J. Invert. Pathol..

[47] Soto M.A., Shervette V.R., Lotz J.M. (2001). Transmission of white spot syndrome virus (WSSV) to *Litopenaeus vannamei* from infected cephalothorax, abdomen, or whole shrimp cadaver. Dis. Aqua. Organ..

